# Editorial: Pharmacology of Plant Polyphenols in Human Health and Diseases

**DOI:** 10.3389/fphar.2022.945033

**Published:** 2022-06-17

**Authors:** Hari Prasad Devkota, Keshav Raj Paudel, Namrita Lall, Michał Tomczyk, Atanas G. Atanasov

**Affiliations:** ^1^ Graduate School of Pharmaceutical Sciences, Kumamoto University, Kumamoto, Japan; ^2^ Headquarters for Admissions and Education, Kumamoto University, Kumamoto, Japan; ^3^ Centre for Inflammation, Faculty of Science, School of Life Sciences, Centenary Institute and University of Technology Sydney, Sydney, NSW, Australia; ^4^ Department of Plant and Soil Sciences, Faculty of Natural and Agricultural Sciences, University of Pretoria, Pretoria, South Africa; ^5^ School of Natural Resources, College of Agriculture, Food and Natural Resources, University of Missouri, Columbia, MO, United States; ^6^ College of Pharmacy, JSS Academy of Higher Education and Research, Mysuru, India; ^7^ Department of Pharmacognosy, Medical University of Bialystok, Bialystok, Poland; ^8^ Ludwig Boltzmann Institute Digital Health and Patient Safety, Medical University of Vienna, Vienna, Austria; ^9^ Institute of Genetics and Animal Biotechnology of the Polish Academy of Sciences, Jastrzebiec, Poland

**Keywords:** polyphenols, flavonoids, bioavailability, oxidative stress, tannins, obesity

Polyphenols are one of the most abundant classes of secondary metabolites in plants and particularly relevant in leafy vegetables, fruits, berries, tea, and other beverages, with a wide range of health-promoting activities reported. They are also among the most widely studied natural products regarding their biosynthesis, chemical properties, and pharmacological activities. Different polyphenols such as anthocyanins, coumarins, carotenoids, flavonoids, and xanthones have been reported to be promising anti-inflammatory, anticancer, antidiabetic, antihyperlipidemic, antioxidant, and neuroprotective agents (Ganesan and Xu, 2017; Khan et al., 2019). In recent years, there is a growing number of papers that deal with the isolation, characterization, and bioactivity evaluation of polyphenols. However, many published results are mostly based on *in vitro* evidence. At the same time, there is less focus on the bioavailability, study of detailed mechanisms of action using animal models, and possible toxicities. There have also been concerns about the specificity of the compounds’ effects and the dose levels needed to achieve such outcomes. Although many polyphenols show potent bioactivity during testing with *in vitro* evaluation systems, there are various challenges at an *in vivo* level. The *in vitro* results often cannot be translated to similar effects in animal models and clinical studies (Hu, 2007).

Our Research Topic “*Pharmacology of Plant Polyphenols in Human Health and Diseases*” aimed to collect studies related to plant polyphenols from characterization to their molecular pharmacology, safety, and bioavailability evaluation studies. A total of six articles were published on this topic, including two original research articles and four review articles.

Pandeya et al. reported the anti-obesity activity of a botanical drugs formulation named 18KHT01, prepared by mixing acorn jelly powder of *Quercus acutissima* Carruth. (Fagaceae), dried leaf buds of *Camellia sinensis* (L.) Kuntze (Theaceae), dried aerial parts of *Geranium thunbergii* Siebold and Zucc. (Geraniaceae) and fruit juice of *Citrus × limon* (L.) Osbeck (Rutaceae), using *in vitro* and *in vivo* studies. In *in vitro* experiments, the 40% ethanol extract of the formulation showed antioxidant and anti-adipogenic activity. In high fat diet-induced obese mice, the extract ameliorated the high fat diet-induced obesity and insulin resistance. Authors suggested that the formulation may also have similar effects in humans. However, detailed clinical studies are necessary to evaluate the efficacy.

Soeng et al. evaluated the vasorelaxant activity of the ethanolic extracts obtained from the brown, green, red, and black rice bran using Porcine coronary artery endothelial cells. Among different extracts, extract obtained from red rice bran (RRBE) showed a strong endothelium-dependent vasorelaxant effect by inducing phosphorylation of eNOS and Src in cultured endothelial cells resulting in stimulation of nitric oxide formation. Further chemical analysis revealed taxifolin as a major compound in the RRBE which also showed vasoprotective activity.

Rudrapal et al. summarized the antioxidant activity of dietary polyphenols and their role in the preventing oxidative stress-related diseases such as diabetes, cancer, cardiovascular diseases, and neurodegenerative and inflammatory diseases. Authors also summarized the detailed molecular mechanisms of antioxidant dietary polyphenols in the prevention of aging, neurodegenerative disorders, cardiovascular diseases, diabetes, cancer, inflammatory diseases, and infectious diseases based primarily on *in vitro* data and animal experiments. Authors concluded by emphasizing the need for clinical studies to establish similar evidence in humans.

Zhao et al. provided a concise review of the biological sources, pharmacological activities, and pharmacokinetic profiles of a flavonoid, alpinetin (7-hydroxy-5-methoxyflavanone). Authors summarized the molecular mechanisms for the reported anticancer, hepatoprotective, cardiovascular protective, antibacterial, and antiviral activities based on *in vitro* and animal studies. Authors also provided details about the ADME profiles of alpinetin, highlighting its poor bioavailability, rapid clearance *in vivo*, and some strategies to enhance its bioavailability such as complexation with cyclodextrins and formulation of novel drug delivery systems using nanoparticles.

Marcinczyk et al. provided a comprehensive update on tannins as hemostasis-modifying agents. Starting with the natural abundance and chemical characteristics of tannins including ellagitannins, gallotannins, and procyanidins, authors summarized the available scientific information on the pharmacokinetic profiles of tannins. The later part of the article describes the effects of tannins on platelet activity, red blood cell membrane elasticity, coagulation system, fibrinolysis system, endothelium physiology, and thrombosis. Authors also highlighted the challenges associated with the low bioavailability of tannins and insufficient *in vivo* and clinical data.

Li et al. summarized the role of resveratrol in the management of sepsis, a life-threatening organ dysfunction syndrome. Being a naturally abundant and highly studied natural polyphenol, resveratrol is well known for its antioxidant, anti-inflammatory, antiviral activities, etc. Authors analyzed the activity of resveratrol from the experimental results obtained with various sepsis models using different organs such as lung, kidney, liver, brain and circulatory system, immune system, and gastrointestinal tract. They concluded that the preliminary studies had provided the evidence for protective effects. However, improving the bioavailability remains a challenge, along with the insufficient clinical studies to explore resveratrol for the therapeutic use in sepsis.

In conclusion, this Research Topic has provided some new experimental data and updated review about the pharmacological activities of polyphenols mainly based the results of *in vitro* activities. Most of the articles published in this topic highlighted the poor bioavailability of polyphenols, its relation to challenges in using animal models, and the lack of clinical evidence. Exploration of the detailed molecular mechanisms of actions of polyphenols’ bioactivities is necessary to develop them as potentially therapeutic and functional/nutritional agents. Detailed studies assessing their potential bioavailability and optimizing therapeutic doses are also essential.
